# Expression of P63 and its correlation with prognosis in diffuse large B-cell lymphoma: a single center experience

**DOI:** 10.1186/s13000-019-0880-7

**Published:** 2019-11-11

**Authors:** Wan-Ming Hu, Jie-Tian Jin, Chen-Yan Wu, Jia-Bin Lu, Li-Hong Zhang, Jing Zeng, Su-Xia Lin

**Affiliations:** 1 Department of Pathology, Cancer Center, Sun Yat-Sen University, Guangzhou, China; 2 State Key Laboratory of Oncology in South China, Cancer Center, Sun Yat-Sen University, Guangzhou, China; 3 Collaborative Innovation Center for Cancer Medicine, Guangzhou, Guangdong China; 4 Department of Pathology, School of Basic Medical Sciences, Southern Medical University, Guangzhou, China

**Keywords:** P63, P40, P53, Ki67, DLBCL, Prognosis

## Abstract

**Background:**

Large B cell lymphoma (DLBCL) is the most common type of non-Hodgkin’s lymphoma among adults. In some cases, DLBCL may seem similar to carcinoma cells, presenting a round, oval, or polygonal shape and clear nuclei. We found that the expression of P63 accounted for a considerable proportion of DLBCL cases. Under the circumstances, P63 expression may lead to a misdiagnosis, especially with a small biopsy. We aim to investigate the expression status and prognostic significance of P63 in a cohort of Chinese DLBCL patients.

**Methods:**

P63, ΔNP63(P40), P53 and Ki67 were detected by immunohistochemistry (IHC). A ROC curve was adopted to find the best cut-off value for positive P63/P53 expression and high Ki67 expression. We defined P53 as positive when ≥50% of the tumor cells showed staining. The relationship between P63 and P53/Ki67 expression was examined. Time-to-event endpoints were estimated according to the Kaplan-Meier method. Moreover, multivariate analyses were conducted to evaluate the prognostic factors in DLBCL.

**Results:**

Out of all the 159 DLBCL cases, 76 (47.8%) expressed P63 in the nuclei, while 41 (25.8%) were determined to have high expression by using a ROC cut-off value “≥6”. Examination of the different P63 isoforms revealed that the ΔNP63(P40) was unclearly and weakly expressed in only 3 cases, showing a fuzzy yellow cytoplasm. P63 expression was not correlated with subtype (GCB or non-GCB) or P53 but was correlated with a high proliferative index (Ki67). Kaplan-Meier analyses revealed that P63 expression was correlated with overall survival, and P63 positive cases showed poor survival outcomes (*P*<0.05) in our cohort.

**Conclusions:**

ΔNP63(P40) is a useful marker in the differential diagnosis of poorly differentiated squamous cell carcinoma versus DLBCL in small needle biopsy. P63 may be involved in DLBCL tumor progression, and it is an unfavorable prognostic marker in DLBCL. A subgroup of P63 and P53 coexpression DLBCL patients with an extremely poor prognosis should be noted.

## Introduction

Diffuse large B-cell lymphoma (DLBCL), the most common form of non-Hodgkin’s lymphoma (NHL), causes 30–40% of NHL in adults. DLBCL patients usually present with tumors in single or multiple lymph nodes or extranodal sites, and the tumors grow rapidly. In all DLBCL cases, the common type accounts for 80–85%, and the rare type, consisting of large B cells, accounts for approximately 15–20%. The WHO classification system classified DLBCL into activated B-cell-like (ABC) and germinal center B cell-like (GCB) but was unable to classify DLBCL according to gene expression characteristics, indicating that DLBCL is a heterogeneous disease. Based on this finding, pathologists, in addition to conducting a preliminary diagnosis, need to conduct a more detailed study of the existing DLBCL subtypes to explore potential markers associated with differences in prognosis for further classification studies.

P63 expression is tissue specific, and it is restricted to epithelial cells and certain subpopulations of basal cells in normal tissues. However, some P63 positive cells can occasionally be seen in the germinal centers of lymph nodes and seem to be related to the development of lymphoma [1–3]. Because of an alternate intronic promoter and alternative splicing, P63 encodes two major different isoforms [4], a full length transactivating (TA) domain (TAP63/P63) and a truncated form without the N-terminus (∆NP63/P40). The function of TAP63/P63 is similar to P53, which can regulate the expression of P53 downstream target genes, block the cell cycle, induce apoptosis, and may be a candidate tumor suppressor. However, ∆NP63/P40 can antagonize P53 or P63 by competitively binding DNA sites, thereby promoting cell proliferation and inhibiting apoptosis, and it functions similar to an oncogene. In a range of human cancers, the expression of P63 was proven to be associated with tumor development. Kenji Hibi et al. [5] found that overexpression of ∆NP63/P40 may lead to increased epithelial stem cell renewal and promote tumor growth in squamous cell carcinoma. However, there were controversial conclusions on the role of P63 and its prognostic significance in lymphoma. A study from Japan showed the expression of the P63 protein in 34% of cases with poor overall survival (OS) [6]. However, a study from America found that a subset of DLBCL (32% of cases) expressed P63, but it did not correlate with overall survival [7]. China has a large number of DLBCL patients, but no study thus far has reported P63 expression in Chinese DLBCL patients. We evaluated P63 expression by immunohistochemistry to determine the expression of P63 and its prognostic significance in a Chinese DLBCL cohort.

## Materials and methods

### Patients

In this study, 211 cases of DLBCL, 8 cases of EBV+ DLBCL, NOS, and 21 cases of primary mediastinal large B-cell lymphoma form SYSUCC (Sun Yat-sen University Cancer Center) occurring between 2004 and 2014 were included. All the clinical data, along with the follow-up data, were obtained from the SYSUCC records or patients’ charts. Oral or written informed consent was acquired in all cases before this study. In 211 cases of DLBCL, 52 cases were lost to follow-up or clinical data, and the loss ratio was 24.6%. The final number of cases was 159. Overall survival (OS) was calculated from the date of diagnosis until death or the last follow-up. The mean follow-up time was 67 months (range: 1 to 156 months).

### IHC (Immunohistochemistry)

Formalin-fixed, paraffin-embedded tissue blocks were cut into slides to detect the protein expression of P63 (Dako), P40 (Dako), P53 (Dako) and Ki67 (Dako) by IHC, using a standard technique demonstrated previously [8]. The adjusted Allred scoring system was applied to evaluate the results of P63 expression; the total value was 0–12 by positive ratio×staining intensity. To be specific, the positive proportion was scored as “1 for 0-25%, 2 for 26%-50%, 3 for 51%-75%, and 4 for >75%”, and the staining intensity was scored as “0 for no staining, 1 for light yellow, 2 for yellowish brown, and 3 for brown”. The ROC curve was used to find the cut-off value of P63/P53 positive expression and Ki67 high expression. Using the ROC curve, we defined the following: P53 was positive when ≥50% of tumor cells showed staining, a positive P63 expression value was determined when “P63 expression≥6”, and Ki67 high expression was determined when “Ki67 ≥ 80%”.

### TCGA dataset

The TCGA (The Cancer Genome Atlas) cohort of 47 DLBCL patients with detailed clinical information was downloaded from the public database (https://tcga-data.nci.nih.gov/tcga/tcgaDownload.jsp) and analyzed with the GEPIA tool [9] (http://gepia.cancer-pku.cn/).

### Statistical analysis

The relationship between P63, P53, and Ki67 expression and clinic-pathological parameters was evaluated by a Chi-square test. The Kaplan-Meier method was used to draw the survival curves, and they were compared by log-rank test. Univariate and/or multivariate Cox regression analyses were used to assess the influence of variables on survival. *P* < 0.05 was defined as significant.

## Result

### P63, P40, P53, and Ki67 expression in DLBCL cases; P63 was expressed in almost half of the DLBCL cases

**Fig. 1:**
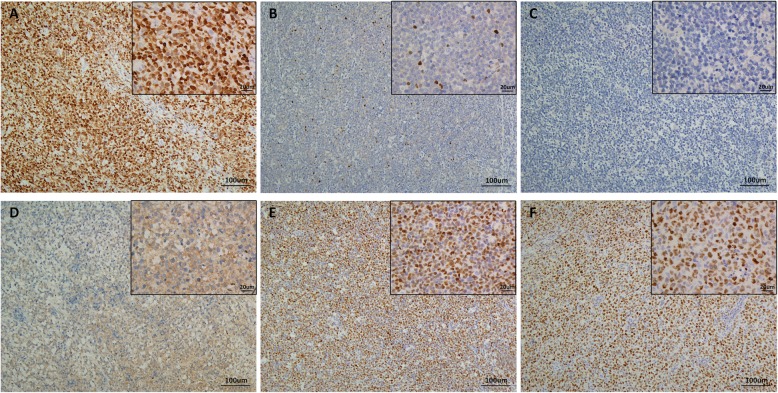
Representative figure of IHC staining with an anti-P63 antibody of strong expression (**a**), week expression (**b**), no expression (**c**), anti-P40 antibody with weak cytoplasm expression (**d**), strong expression of anti-P53 antibody (**e**) and high Ki67-index (**f**)

**Fig. 2:**
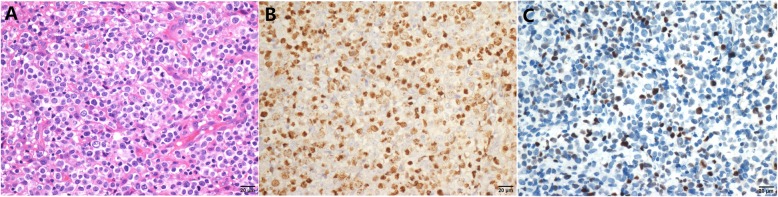
P63 expression in EBV+ DLBCL. **a** HE staining of EBV+ DLBCL. **b** EBV in situ hybridization showed positive results. **c** P63 expression score was “6” in this case, for the proportion was “2 for 26%-50%” and staining intensity was “3 for brown”

**Fig. 3:**
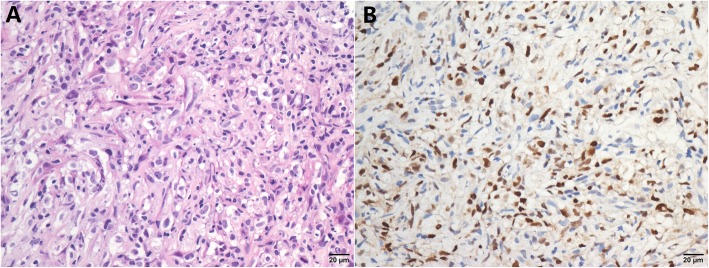
P63 expression in primary mediastinal large B-cell lymphoma. **a** HE staining of a typical case of primary mediastinal large B-cell lymphoma. **b** P63 was highly expressed in this case, with 9 scores (3 for proportion * 3 for staining intensity)

We performed P63, P40, P53 and Ki67 IHC in all 159 DLBCL cases with clinical information and follow-up data. These four antibodies were expressed in the nuclei (Fig. 1). An additional 8 cases of EBV+ DLBCL and 21 cases of primary mediastinal large B-cell lymphoma were tested for P63 (Figs. 2 and 3). In the EBV+ DLBCL cases, 4/8 (50%) cases expressed P63 in the nucleus, while 2/8 (20%) cases had high expression using a cut-off value of “6”. In primary mediastinal large B-cell lymphoma cases, 21/21(100%) cases expressed P63 in the nuclei, while 17/21 (81%) cases had high expression using a cut-off value of “6”. In all 159 DLBCL tissues examined, 76 (47.8%) cases expressed P63, while 41 (25.8%) cases had high expression using a cut-off value of “6”. Notably, ΔNP63(P40) was unclearly and weakly expressed in only 3 cases with a fuzzy yellow cytoplasm, and we considered it as a false positive from the background (0%). P53 was positive in 39 (24.5%) cases using the cut-off value of “≥50% tumor cells showed staining”. There were 88 cases (55.3%) that exhibited a high proliferative index with Ki67 ≥ 80%.

### P63 expression and correlation with clinical-pathological characteristics of the patients

As shown in Table 1, no significant correlation was found between P63 expression and clinical parameters, including age, sex, stage, LDH level, subtype and P53 (*P* > 0.05), with the exception of Ki67 (*P* = 0.021).

**Table 1:** Clinical-pathological characteristics of the patients and P63 expression

	No. of cases	P63		P
		Negative	Positive	
Age				0.397
<60	98	75	23	
≥ 60	61	43	18	
Gender				0.852
Male	95	70	25	
Female	64	48	16	
Stage				0.347
I + II	67	45	22	
III + IV	92	73	19	
LDH level				0.458
Normal	90	69	21	
High	69	49	20	
Subtype				0.537
GCB	88	67	21	
Non-GCB	71	51	20	
P53				0.097
<50%	120	93	27	
≥ 50%	39	25	14	
Ki67				0.021
<80%	71	59	12	
≥ 80%	88	59	29	

### P63 expression and overall survival

**Fig. 4:**
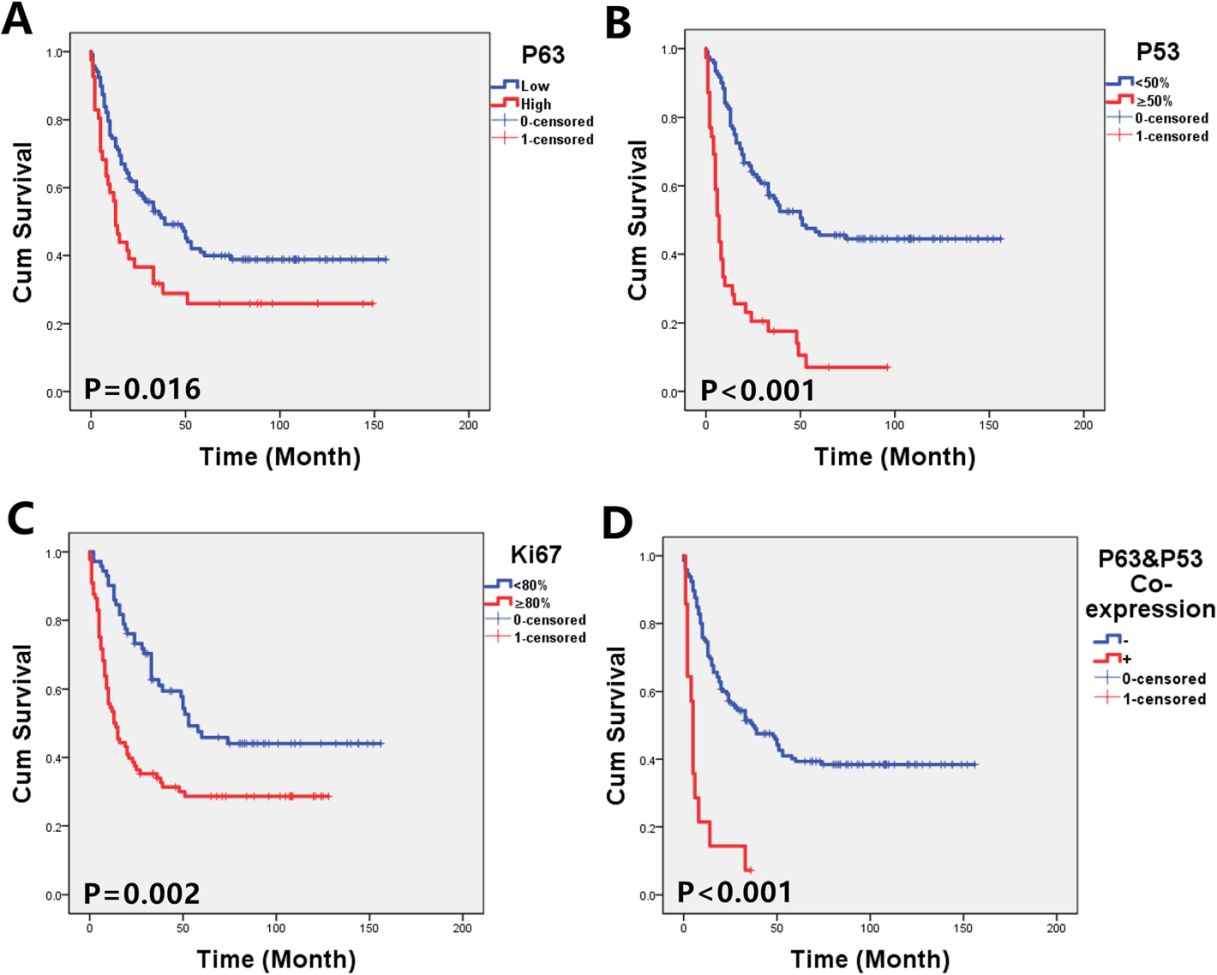
OS curves in the subtypes of DLBCL. **a** Comparison of P63 high-expression (score ≥ 6) and low-expression (score<6) groups. **b** Comparison of P53 positive (≥50%) and negative (<50%) groups. **c** Comparison of Ki67 high expression (≥80%) and low expression (< 80%) groups. **d** P63 and P53 coexpression cases showed the worst prognosis

The median survival time of the P63 negative group (39 months) was significantly longer than that of the P63 positive patients (13 months) (*p* = 0.016). Our data also show a significant correlation between P53 expression and OS. There was a median follow-up of 51 months for P53 negative cases and only 7 months for P53 positive cases (*p* < 0.001). The same results were also observed for Ki67, with 10 months in the Ki67 high expression cohort to 48 months in Ki67 the low expression cohort (*p* < 0.001). Further analysis indicated that P63 and P53 coexpression cases (14 cases) had the worst prognosis, with a median survival time of only 5 months. The results are shown in Fig. 4. Moreover, a multivariate Cox analysis was used to assess the potential prognostic factors (Table 2). Variables, including sex, age, stage, LDH, type, P53, Ki67 and P63 expression, were independent factors.

**Table 2:** Multivariate analysis for overall survivals

Variable	HR (95% CI)	*p* Value
Sex (female VS male)	1.150 (0.768–1.724)	0.497
Age (<60 VS ≥60)	1.663 (1.095–2.527)	0.017
Stage (I II VS III IV)	3.682 (2.321–5.673)	< 0.001
LDH (low VS high)	2.580 (1.634–3.753)	< 0.001
Type (GCB VS non-GCB)	1.606 (1.066–2.420)	0.023
P53 (<50% VS ≥50%)	3.355 (2.156–5.221)	< 0.001
Ki67 (<80% VS ≥80%)	1.981 (1.292–3.037)	0.002
P63 (Negative VS Positive)	1.666 (1.076–2.580)	0.022

*HR* hazard ratio, *CI* confidence interval

### P63 is highly expressed in DLBCL and associated with poor prognosis in TCGA datasets

**Fig. 5:**
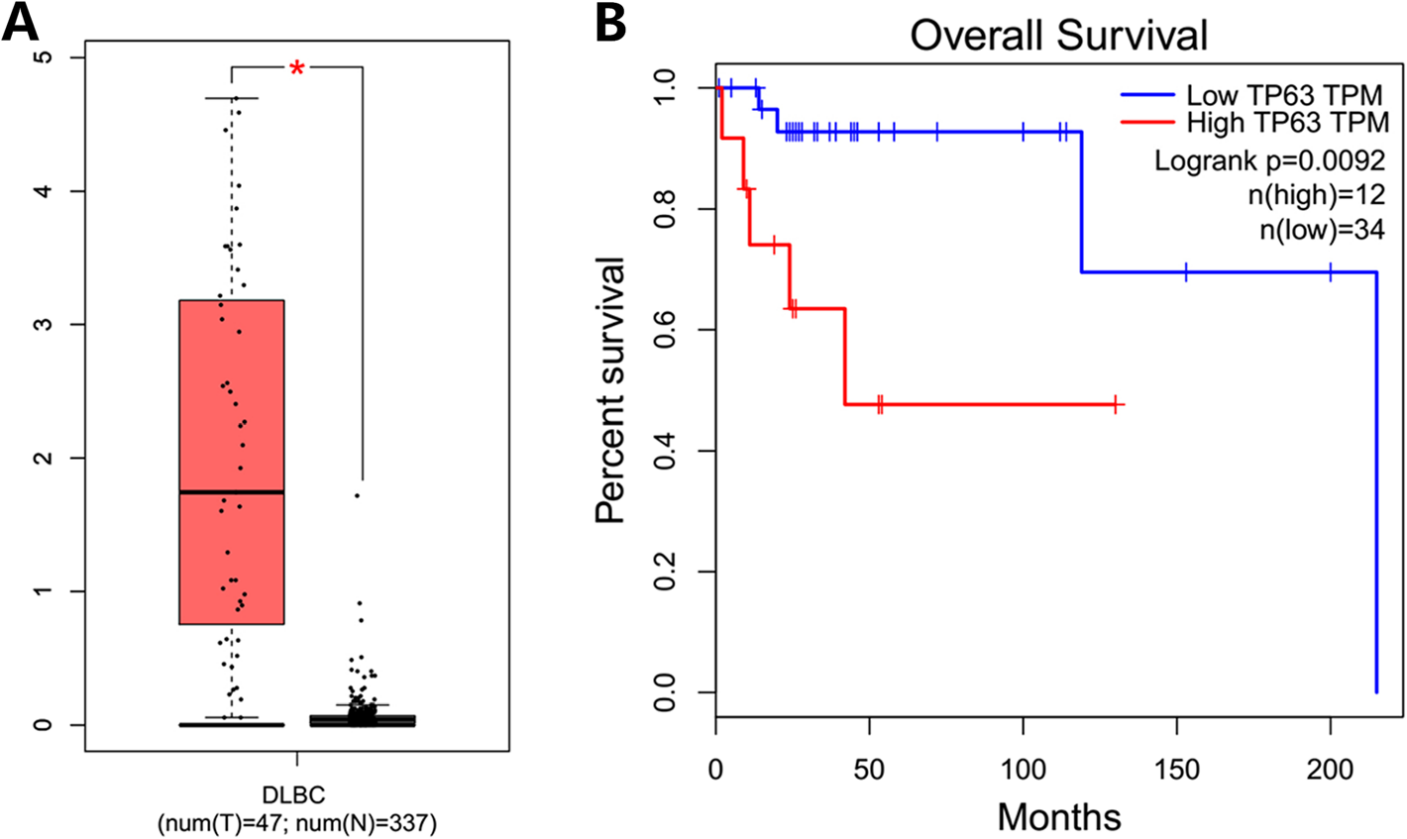
**a** The expression intensity of P63 mRNA was significant (*P* < 0.001) between DLBCL and normal lymphoid tissues, and **b** predicted unfavorable prognosis in TCGA DLBCL datasets (*P* = 0.0092)

To further confirm our results, we queried P63 expression in the TCGA datasets of DLBCL patients and normal lymphoid tissues. P63 mRNA was highly expressed in 12 DLBCL cases (12/47, 25.5%), and the expression intensity of P63 mRNA was significantly different (*P* < 0.001) between DLBCL and normal lymphoid tissues, with a fold change greater than 2. High expression of P63 was also an adverse prognostic factor of DLBCL in TCGA (Fig. 5).

## Discussion

P63, an important transcription factor, was discovered in 1998 and is located on chromosome 3q27–28. The P63 gene has structural and functional homology with the P53 gene family, regulating downstream target genes, activating various signaling pathways, and participating in the regulation of a variety of biological functions. P63 is at the key node of the regulation network, involved in mechanisms of tumorigenesis and development, such as cell cycle regulation, apoptosis, differentiation and cell adhesion and migration. It is well known that P53 is usually a tumor suppressor gene, but many studies have found that P63 may promote tumor development in human primary tumors and cell lines.

DLBCL (diffuse large B-cell lymphoma) exhibits clinical heterogeneity and responds differently to treatment and prognosis. Although survival rates can be estimated based on clinical parameters, recent literature reports that a group of tumor suppressor proteins and oncogenic proteins are associated with prognosis [10]. However, at present, there are contradictory results about the prognostic significance of P63 in lymphoma, especially in DLBCL. In addition, in our daily pathology work, especially in some needle biopsy cases, DLBCL may mimic carcinoma cells, presenting a round, oval, or polygonal shape and clear nuclei that are positive for P63, and we found that P63 is expressed in a considerable proportion of DLBCL. Under the circumstances, it may easily be misguided. In our cohort, we found there was no P40 (a specific marker of squamous cell carcinoma) expression in DLBCL, which may be extremely useful for the differential diagnosis of poorly differentiated squamous cell carcinoma versus DLBCL, especially in small sample needle biopsies.

P63 is a particularly useful marker in the differential diagnosis of lymphoma as well, with a high positive predictive value of 96% for primary mediastinal large B cell lymphomas, but very rare in CHL (classical Hodgkin’s lymphoma) [11]. Zamò A et al. [12] also found that P63 was a useful diagnostic marker of primary mediastinal large B-cell lymphoma at both the protein and mRNA levels. Shi QY et al. [13] used immunohistochemical methods to show that tumor cells of mediastinal large B-cell lymphoma were highly positive for P63 (84%, 16/19), and their results were consistent with ours (81%, 17/21). However, few studies have investigated the expression and prognosis of P63 in DLBCL. In 2002, Di Como et al. [2] found a P63-positive population in non-Hodgkin’s B-cell lymphoma and normal lymph nodes and detected the expression of P63 in peripheral blood lymphocytes by Western blot. In addition, P63 expression levels are higher in tumor lymphocytes compared to normal lymphoid tissue. However, they did not explore the prognostic significance of P63 positive cases. In a study regarding 64 primary cutaneous large B cell lymphomas, Alistair Robson et al. [14] demonstrated that the expression of P63 protein was significantly increased in pc DLBCLL (70%) compared to pc FCCL (12%) (*P* < 0.001). Evaluation by Ki67 immunostaining revealed that high expression of P63 was associated with a higher proliferation index (*P* = 0.015). However, when analyzing the current data, they did not find a significant association between P63 expression and patient outcome. Hedvat et al. [7] demonstrated that 32% of cases expressed P63 in the nuclei of DLBCL cells, and P63 expression is associated with the proliferation index; they also concluded that P63 did not correlate with overall survival. Interestingly, after that, conflicting results regarding P63 expression and its prognostic role in DLBCL were published. In 2005, Park CK et al. [15] found that P63 was expressed in 32/61 cases (52.5%) of DLBCL, including 15 strong expression cases in their cohorts, and it was a significant poor survival factor in DLBCL (*p* = 0.0228). Fukushima et al. [6] investigated and evaluated the expression pattern of P63 in B-cell lymphomas and found that P63 was expressed in the nuclei of tumor cells in 22 of 65 (34%) DLBCL cases, and they also reported that P63 is a disadvantageous factor for prognosis in DLBCL. However, although Hallack et al. [16] observed P63 expression in 15.1% of DLBCL cases, no correlation was found between P63 expression and OS (*p* = 0.09). Furthermore, they proposed that being P63(+) provided a protective effect on high-intermediate and high risk DLBCL, and DLBCL patients with P63(+) have a better DFS than negative cases [16]. A recent study concerning high-risk diffuse large B-cell lymphoma also reported that P63 expression confers significantly better survival outcomes [17].

In the present study, we characterized the expression of P63 in a series of 159 Chinese DLBCL cases and found that 76 (47.8%) cases were P63 positive regardless of the intensity and percentage, and the results were completely consistent with studies from Japan and Korea. In addition, we found that 4/8 (50%) EBV+ DLBCL cases expressed P63 in the nucleus, while in 2/8 (20%) cases, it was highly expressed. Although the sample size was small, the results were almost the same as DLBCL in our cohort, indicating that EBV status had no effect on P63 expression. At the same time, we also discovered that P63 positive patients had a worse prognostic value, which was inconsistent with previous studies based on non-Asian populations. We think a possible cause of the inconsistent findings is ethnic differences because all of the reports of poor prognosis were Asians. This conclusion needs further research and confirmation with large samples. Other possible reasons might be technical factors related to staining quality, cut-off value, data interpretation, treatment regimens and follow-up time. For instance, Hallack et al. [16] used a 50% cut-off to indicate immunohistochemical positivity. In our study, a cut-off value of “≥6” was used based on the adjusted Allred scoring system and ROC curve, and this difference should be tested in other studies to confirm its repeatability.

Furthermore, we did not observe a significant correlation between P63 expression patterns and subgroups of GCB-like DLBCL and non-GCB DLBCL, which is consistent with the research of Hallack [16]. We discovered that P63 expression was correlated with the Ki67 proliferative index in DLBCL, and P63 gene amplification has been reported to correlate with the Ki67 proliferative index in lung cancer [18]. Miller et al. [19] reported that a tumor proliferation index of > 80% was associated with poorer survival in previously untreated patients with aggressive NHL, and we also confirmed this correlation in our DLBCL cohort. Several reports [20–24] have revealed that P53 mutations and P53 overexpression are common events in DLBCL. Their findings were very consistent, as P53 abnormalities were associated with a poor prognostic indicator. In addition, P53 expression in ≥50% of lymphoma cells was used as a cut-off value, which is consistent with our study. In our combined analysis, it was found that the prognosis for P63 and P53 co-expression cases is extremely poor. Whether these DLBCL patients need radical treatment still requires further research in the future.

## Conclusion

In conclusion, P63 is expressed in almost half of the DLBCL cases, and it appeared to have a negative effect on survival in Asians. ΔNP63(P40) is useful marker in the differential diagnosis of poorly differentiated squamous cell carcinoma versus DLBCL. Moreover, a subgroup of P63 and P53 coexpressing DLBCL patients with the worst prognosis should be noted. Targeting P63 expression and function may be a novel therapeutic strategy for particular subgroups of DLBCL patients.

## Data Availability

There are no additional supporting data available.

## Abbreviations

CHL: Classical Hodgkin’s lymphoma
CI: Confidence interval
DLBCL: Large B cell lymphoma
HR: Hazard ratio
IHC: Immunohistochemistry
NHL: non-Hodgkin’s lymphoma
OS: Overall survival
SYSUCC: Sun Yat-sen University Cancer Center
TCGA: The Cancer Genome Atlas
